# Deep Eutectic Solvent-Based Dispersive Liquid–Liquid Microextraction Coupled with LC-MS/MS for the Analysis of Two Ochratoxins in Capsicum

**DOI:** 10.3390/molecules28227634

**Published:** 2023-11-16

**Authors:** Hongbo Yang, Jin Li, Jianfei Mao, Chan Xu, Jieyu Song, Feng Xie

**Affiliations:** 1 School of Public Health, The Key Laboratory of Environmental Pollution Monitoring and Disease Control, Ministry of Education, Guizhou Medical University, Guiyang 561113, China; jinliessay@163.com (J.L.); xuchan0712@163.com (C.X.); ajieyusong@163.com (J.S.); xiefeng@mtxy.edu.cn (F.X.); 2 College of Chemistry, Sichuan University, Chengdu 610064, China; 3 Guizhou Jiandee Technology Co., Ltd., Guiyang 550025, China; 4 Guizhou Academy of Testing and Analysis, Guiyang 550014, China; 5 Department of Food Science and Engineering, Moutai Institute, Renhuai 564507, China

**Keywords:** deep eutectic solvent, dispersive liquid–liquid microextraction, ochratoxins, capsicum, green analytical chemistry

## Abstract

Ochratoxins, a common class of mycotoxin in capsicum, and techniques and methods for the determination of mycotoxins in spices have been increasingly developed in recent years. An innovative and eco-friendly method of dispersive liquid–liquid microextraction (DLLME) was demonstrated in this study, based on a synthesized deep eutectic solvent (DES) combined with LC-MS/MS, for the quantification and analysis of two ochratoxins in capsicum. The DES-DLLME method parameters entail selecting the DES type (thymol:decanoic acid, molar ratio 1:1) and DES volume (100 μL). The volume of water (3 mL) and salt concentration (0 g) undergo optimization following a step-by-step approach to achieve optimal target substance extraction efficiency. The matrix effect associated with the direct detection of the target substance in capsicum was significantly reduced in this study by the addition of isotopic internal standards corresponding to the target substance. This facilitated optimal conditions wherein quantitative analysis using LC-MS/MS revealed a linear range of 0.50–250.00 µg/mL, with all two curves calibrated with internal standards showing correlation coefficients (r^2^) greater than 0.9995. The method’s limits of detection (LODs) and limits of quantification (LOQs) fell in the ranges of 0.14–0.45 μg/kg and 0.45–1.45 μg/kg, respectively. The method’s spiked recoveries ranged from 81.97 to 105.17%, indicating its sensitivity and accuracy. The environmental friendliness of the technique was assessed using two green assessment tools, AGREE and complexGAPI, and the results showed that the technique was more in line with the concept of sustainable development compared to other techniques for detecting ochratoxins in capsicum. Overall, this study provides a new approach for the determination of mycotoxins in a complex food matrix such as capsicum and other spices using DES and also contributes to the application of green analytical chemistry methods in the food industry.

## 1. Introduction

Peppers (*Capsicum annuum* L.) are one of the world’s most widely used spices, which are included in hundreds of recipes and also served as separate foods in restaurants and households [1]. However, the extensive use of peppers has resulted in various causes and forms of contamination, posing a significant threat to human health [2]. Peppers may become polluted by a variety of causes throughout the process from farm to table. For instance, the use of fertilizers during the planting process can lead to heavy metal pollution [3]. Additionally, there is a risk of pollution due to the illegal addition of substances like Rhodamine B [4], whilst the improper processing and storage can also result in microbial contamination [5] and mycotoxin contamination [6]. These pollutants not only cause economic losses but also present potential hazards to human health. 

Mycotoxin pollution poses a significant threat to human health compared to other sources of pollution in capsicum [7]. Mycotoxin is a general term for a class of secondary metabolites produced by various types of fungi [8]. The mycotoxins that contaminate chili peppers mainly come from aflatoxins and ochratoxins (OTs) produced by Aspergillus, Penicillium, Trichoderma spp., and other fungi [8]. OTs are mainly secondary metabolites produced by *Aspergillus ochraceus*, *Aspergillus carbonarius*, and *Aspergillus oryzae*. The OTs family can be divided into ochratoxin A, ochratoxin B, ochratoxin C, ochratoxin α, etc., of which ochratoxin A is the most toxic and widely distributed [9]. Several studies have shown that ochratoxin A (OTA) has a variety of toxicological effects, such as teratogenicity, carcinogenicity, hepatotoxicity, reproductive toxicity, etc. [10]. Therefore, OTA has been categorized as a group 2B cancer compound by the International Agency for Research on Cancer (IARC) [11]. Ochratoxin B (OTB) is a kind of non-chlorinated form of OTA, with the main difference being that it does not contain chlorine in its molecular structural formula. Compared with OTA, OTB is considered to have relatively low toxicity [12].

In recent years, certain standards or regulations limiting the content of OTA in food have been formulated in different countries, regions, and organizations, in order to prevent the harm of OTA to human health. The Chinese National Food Safety standard (GB2761-2017) [13] specified the OTA limit values in coffee, coffee beans, and wine. In the European Union, EU 2015/1137 [14] stipulates that the amount of OTA in capsicums should not exceed 15 μg/kg.

In recent years, there have been advancements in technology for identifying OTs in food. These advancements include liquid chromatography–tandem mass spectrometry (LC-MS/MS), thin layer chromatography (TLC), and high-performance liquid chromatography with fluorescence detector (HPLC-FLD) [15,16,17]. In addition, immunoaffinity chromatography [18,19] by specific binding between antibodies and antigens during pre-treatment has been utilized for the detection of OTs in complex foods, and the enzyme-linked immunosorbent assay (ELISA) [20,21] has been widely used for the detection of OTs in food matrices. Although these techniques and methods provide accurate and reproducible results, it should not be overlooked that they generally use hazardous volatile organic solvents, such as acetonitrile or methanol, which are well-known environmental pollutants and are hazardous to humans and the environment due to their high toxicity and poor biodegradability. With the introduction of the concept of green analytical chemistry (GAC) [22], and in order to meet society’s growing demand for more sustainable products, these solvents should be avoided as much as possible.

Atom economy [23] is one of the most popular evaluation metrics in green analytical chemistry, and atom economy-compliant green solvents are growing to replace traditional solvents. Deep eutectic solvents (DESs) are currently the most promising green solvents that meet the atomic economy [24]. Since the concept was introduced by Abbot’s group in 2001 [25], it has been widely used to extract target analytes from different matrices. DES has a simple composition, and in a brief overview, it generally consists of two substances, one of which acts as a component of the hydrogen bond donor (HBD) and the other is known as the hydrogen bond acceptor (HBA), which are bonded to each other by hydrogen bonding [26]. Compared with ionic liquids (ILs), DES are economical, non-toxic, easy to prepare, biocompatible, and biodegradable, making them the solvents of choice for green analytical chemistry [27], and DESs are gradually being utilized in many aspects [28,29,30,31,32] of analytical testing.

Inspired by the work published by Azza H. Rageh’s group [33]. In this study, we synthesized DESs with thymol as HBD and decanoic acid as HBA and applied it for the first time for the detection of OTs in capsicum and optimized the whole extraction process. The types of DESs were determined to be decanoic acid and thymol, their molar ratio was determined to be 1:1, the volume of DES was 100 μL, the volume of water added was 2 mL, and the addition of salt was not required; moreover, it was briefly characterized by infrared, and finally, the method was evaluated for its green chemistry using a complex GAPI with AGREE.

## 2. Results

### 2.1. Characterization of DES Composition

**Figure 1:**
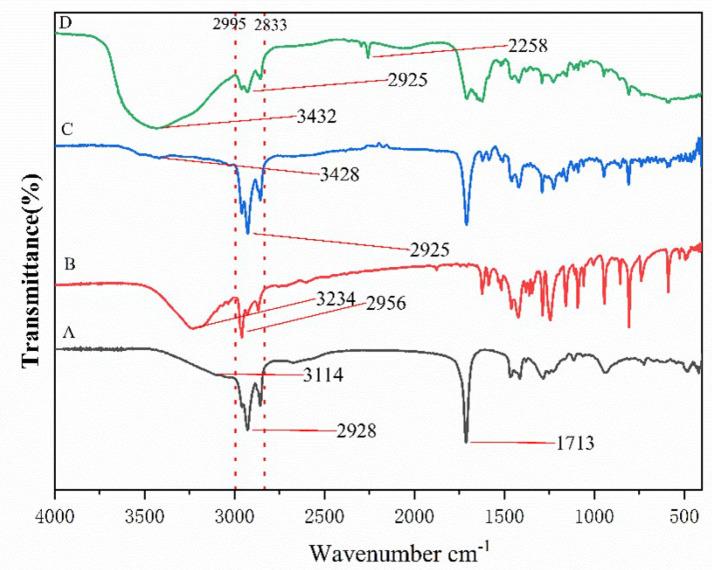
FT-IR spectra of decanoic acid (**A**), thymol (**B**), DES (THY: DecA1:1) before the reaction (**C**), and DES (THY:DecA1:1) after the reaction (**D**).

DESs synthesized with different compositions and molar ratios of HBAs and HBDs were shown in Table 1. The FT-IR spectra of thymol, decanoic acid, DES (THY-DA 1:1) before the reaction and after the reaction were recorded to study the interaction between the components as given in Figure 1.

In the FT-IR spectrum of thymol, the retching vibrations of the -CH_3_ and -CH_2_ groups in the region from 2995 to 2833 cm^−1^ are evident, and the -OH stretching vibration band stretching vibration band appears as a characteristic peak at 3234 cm^−1^. On the other side, regarding the FT-IR spectrum of decanoic acid, the vibrations of -CH_3_ and -CH_2_ at 2995 to 2833 cm^−1^ are also evident, and the -C=O stretching band of decanoic acid appears at 1713 cm^−1^. The FT-IR spectrum of the deep eutectic solvent showed that it possessed both the contraction vibration of the -CH_3_, -CH_2_ group as well as the contraction vibration of -C=O, and at the same time, the contraction vibration of -OH shifted from 3234 to 3428 cm^−1^, which might be due to the formation of the intermolecular hydrogen bonding, and further proved the successful synthesis of the DES. On the other hand, the FT-IR figure of the DES after participation in the reaction showed an increase in the width of the -OH contraction peak at 3432 cm^−1^ compared with that of the DES before participating in the reaction. In addition, a contraction peak of -CN appeared at 2258 cm^−1^, which indicated that some of the moieties of the DES were changed after participating in the reaction, and some of the acetonitrile solution was solubilized in the phase of the DES after participation in the reaction. Obviously, the differences between the DES synthesized and the reacted DES in this study, as well as the precursors used to synthesize it, mainly appeared in the obvious changes between the characteristic peaks, especially the change in the intensity and position of the contraction peaks of -OH.

### 2.2. DES-DLLME Optimization

In this work, in order to obtain the optimal conditions for the extraction of OTs in capsicum, which is necessary to establish the conditions that produce the best performance of the proposed extraction protocol, the different important variables that affect the extraction efficiency (type of DES, volume of DES, volume of water, and ionic strength, etc.) were discussed in a step-by-step strategy, and all the experiments were repeated three times.

#### 2.2.1. Type of DES

**Figure 2:**
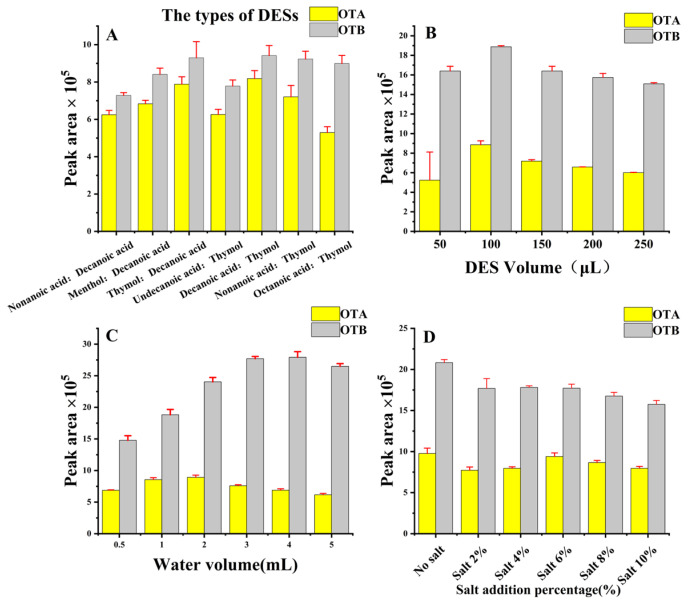
Effect of DESs types (**A**), DES volume (**B**), water volume (**C**), and salt addition percentage (**D**).

The selection of DES is a key part of DLLME that affects the extraction efficiency. The synthesis of different HBDs, mainly medium-chain fatty acids with HBAs such as thymol was tested. In this case, some of the combinations initially synthesized were shown to be clear liquids. However, some of these compounds were found to form white dense precipitates attached to the bottom of the vials after a period of time (greater than 24 h). All of them formed a white dense precipitate attached to the bottom of the bottle, and the formation of these compounds could not be proved to be synthesized as DESs, whereas at a molar ratio of medium chain fatty acids to thymol of 1:1, the state of the clarified liquid was sustained at room temperature, which indicated that these compounds belonged to the successfully DES. Therefore, the series of DES synthesized with a molar ratio of medium chain fatty acids to thymol of 1:1 were chosen to perform DLLME, as well as some DES with thymol as the HBD as a model for examining the extraction of OTs from capsicum. The validation of the above series of DESs was carried out by examining the ratios of the peak area of OTs with the corresponding isotopic internal standard to counteract the complex matrix effect of capsicum. It was found that, for the system under study, the DES with a combination of 1:1 ratio of decanoic acid and thymol gave the best extraction recovery and was selected for further experiments. The effects of DES types was illustrated in Figure 2A.

#### 2.2.2. Effect of DES Volume

The volume of the DES is one of the most important factors considered in the DLLME procedure that affects the extraction efficiency. There is a balancing selection between minimizing the use of extraction solvents for green analysis and maximizing the volume of DES for extraction efficiency. In this work, DES addition volumes ranging from 50 to 250 μL were examined and each experiment was performed in triplicate. The analytical signal (expressed as peak area) increased from 50 μL until it peaked at 100 μL, and along with the continued increase in the volume of DES, a dilution effect occurred, leading to a gradual decrease in the analytical signal from 150 μL. Thus, a DES volume of 100 μL had the best extraction efficiency in this study, as shown in Figure 2B. In addition, the addition of a lower volume of DES, such as 50 μL, would result in a large error and difficult operation in practice, so the volume of DES was chosen to be 100 μL and the subsequent experiments were carried out with the volume selected for further experiments.

#### 2.2.3. Effect of Water Volume

To achieve a higher extraction efficiency, another important involvement, the ratio between acetonitrile and aqueous phase, needs to be examined. The volume of the acetonitrile extract is fixed at 1 mL and varying the volume of the aqueous phase can significantly affect the extraction efficiency of DES. An effort should be made to achieve the maximum possible DLLME efficiency. The effect of different volumes of the aqueous phase on the extraction efficiency was investigated with a constant volume of the acetonitrile phase, and the peak area was used as an evaluation parameter, and each experiment was performed in triplicate. As shown in Figure 2C, OTA had the best extraction efficiency as the volume of the aqueous phase was increased to 2 mL, and then continued to increase the aqueous phase, the extraction efficiency of OTA began to decrease, while OTB had the best extraction efficiency when the volume of the aqueous phase reached 3 mL, and then began to decrease. Considering that the detection of OTA in OTs has more important practical significance than OTB, the volume of the aqueous phase was comprehensively chosen to be 2 mL.

#### 2.2.4. Effect of Salt Addition

Eventually, the addition of salt may affect the ionic strength and thus the extraction efficiency. For the purpose of assessing the possible salting out effects in this study, the commonly used NaCl was considered as the salting out agent, and the effect of salt addition from 0 to 10% (*w*/*v*) on the extraction efficiency was examined, and each experiment was performed three times. As shown in Figure 2D, the addition of salt does not improve the extraction efficiency and even leads to a decrease in the extraction efficiency. Therefore, no salt was added in the experiments, which also reduces the reagent consumption and leads to greener methods.

### 2.3. Method Validation

Initial experimental results showed that there was a pronounced matrix inhibition effect for both OTA and OTB. In order to mitigate the influence of the matrix effect on the experimental results, according to the limit recommendation for OTA in capsicum formulated by EU 2015/1137, three different concentration levels (low, medium, and high) (0.1–20 μg/kg) were added through sample injection and labeling the target analyte and fixed concentration of 10 μg/kg. Standard curves corrected for isotopic internal standard were plotted using the ratios of different concentrations of the target analytes to the isotopic analytes at 10 μg/kg as the independent variables and the ratio of their peak areas as the dependent variable (in triplicate). The coefficient of determination (R^2^) values of OTA and OTB in chili substrate with acetonitrile as solution were higher than 0.9991 and 0.9997, respectively, confirming the linear regression model fitting experimental data. This information, as well as the study concentration range in each evaluation matrix, are shown in Table 2.

In chemical analysis, the matrix refers to the components in the sample other than the analyte of interest, and matrix effects (ME) are mainly caused by the co-elution of matrix components, which should not be ignored when performing method validation [34]. Briefly, ME mainly includes matrix enhancement and matrix attenuation effects, and there are many factors causing matrix effects, which generally include the mass spectrometry separation effect, the type of ionization, the amount and type of sample matrix, and the sample preparation procedure, which in turn affect the quantitative results. Due to the presence of a complex matrix in capsicum [35], which is rich in a variety of vitamins, minerals, amino acids, and alkaloids, a strong matrix effect exists. In this study, the ME was calculated by the following equation:

ME (%) = (K_matrix_/K_s_ − 1) × 100%

K_matrix_: Slope of the standard solution curves prepared using the corresponding matrices. K_s_: Slope of the curve for standard solutions prepared with solvents.

**Figure 3:**
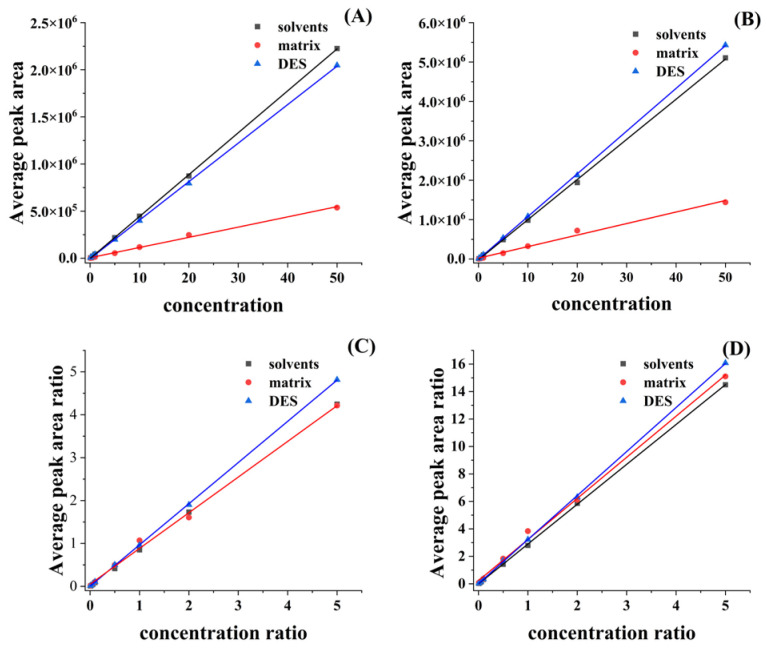
Slopes of OTA and OTB in different matrices, (**A**,**B**): standard curves of OTA and OTB in the solvent, matrix, and DES, respectively: (**C**,**D**): standard curves of OTA and OTB in isotope-corrected solvent, matrix, and DES, respectively.

In this study, the matrix effect between the capsicum extract and the solvent, as well as between the DES involved in the extraction and the solvent, was evaluated in the detection of OTA and OTB. The standard curves evaluated were set up at seven concentration levels (from 0.1 to 50 ng/mL); each experiment was repeated three times; and the results are shown in Table 2. The corresponding standard curves are shown in Figure 3.

From the above results, it can be seen that, when a series of standard curves at different concentration levels are plotted in a general way using the standards of OTA and OTB, the matrix effect is evaluated by the aforementioned method, and it is found that a serious matrix effect, mainly matrix inhibition (ME > 70%), occurs, and that when the actual samples were analyzed by such a standard curve method, the obtained results will be considerably biased. Hence, in order to attenuate the matrix effect on the assay results in this study, the addition of isotopic internal standard correction was used to attenuate the matrix effect of OTA and OTB in the matrix, and the results showed that the matrix effect of OTA was reduced from −75.63% to −2.22%, and that of OTB was reduced from −71.29% to 3.16%, and that the standard curves corrected for internal standards could be directly used to evaluate OTA and OTB in the chili matrices. Although the use of internal standard correction also increased the matrix effect of OTA and OTB in DES, their matrix effects were still within the acceptable range (ME < 20%).

Finally, the LOQs of 1.45 μg/kg and LODs of 0.45 μg/kg for the OTA of the chili pepper matrix in the study; and LOQs of 0.45 μg/kg and LODs of 0.15 μg/kg for the OTB were determined by using the three-fold signal-to-noise ratio (S/N) as the limit of detection (LOQ), and the ten-fold S/N as the limit of quantification (LOQ) for the method, as shown in the details of Table 3.

### 2.4. Application in Analysis of Real Samples

In order to demonstrate the applicability of the developed method, the practical application of this method was carried out on 30 actual capsicum samples from the market of Guiyang City, which were processed as per DLLME of 3.3. It was found that, among the 30 actual capsicum samples, OTA was detected in two capsicum samples and OTB was detected in four capsicum samples., among which two samples containing both OTA and OTB were detected, which demonstrates that the method can be applied to the examination of actual samples. Information on samples with detectable results was summarized in Table 3.

### 2.5. Method Greenness Assessment

**Figure 4:**
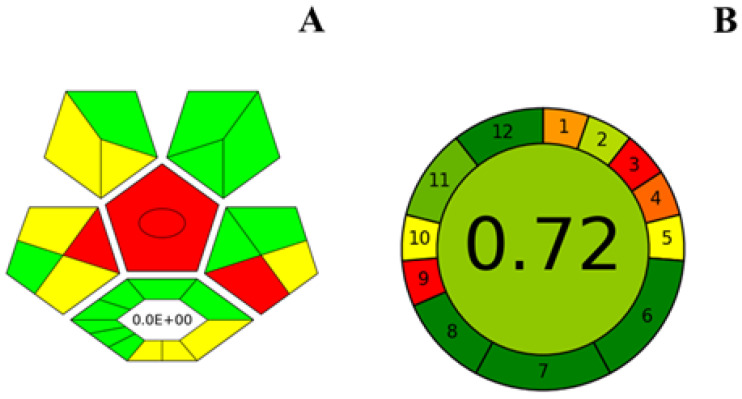
Result of the complexGAPI (**A**) and AGREE (**B**) analysis of the DES-DLLME-HPLC-MS/MS methodology.

Green assessment methods were used to assess the sustainability of various DES-based processes [36]. To evaluate the greenness and safety of DES-based microextraction techniques combined with LC-MS/MS methods, two widely used green assessment tools were selected, including the AGREE (Analytical Green Calculator) tool and the complex GAPI (Green Analytical Procedure Index) based on the GAPI (Green Analytical Procedure Index) improvement. The green evaluation results are shown in Figure 4. AGREE is a quantitative evaluation tool based on the 12 principles of GAC (Green Analytical Chemistry), which was firstly proposed in 2020 [37], it is comprehensive, flexible, and direct; as shown in the figure, the score obtained by the method of this study is 0.72, which indicates that the method of this study is in line with the basic principles of Green Analytical Chemistry.

On the other hand, complexGAPI [38] is also one of the more widely used types of green evaluation tools. Briefly, it has the characteristics of covering the whole stage, which was improved by its founder Justyna Płotka-Wasylka’s group based on the GAPI [39] with the addition of an extra hexagon at the bottom, which is represented in different colors, including from green, yellow, to red, which figure in the five main aspects, namely sample collection, transportation, preservation, storage-to-sample preparation, and the final analysis of all stages of the analytical method. In addition to these aspects, it also includes processes performed prior to the general analytical method. The complexGAPI metric extends the pictogram created for GAPI by adding an additional hexagonal field at the bottom. This field corresponds to the “green” character of the pre-analyzed process. It provides a more comprehensive evaluation of the methodology’s compliance with the green principle. AGREE emphasizes the sustainability of the entire chemical treatment process, while complexGAPI emphasizes the assessment of more macro aspects such as the green economy and environmental hazards. As shown in Figure 4, the resulting pictogram reflects the greenness of the synthesis procedure used to prepare DES and the greenness of the corresponding LC-MS/MS method.

### 2.6. Comparison of Various Methods for Detecting OTs

The comparison of the developed DES-DLLME-LC-MS/MS method with other established methods for the determination of OTs in different food matrices is shown in Table 4. The results indicate that the detection limit and linear range obtained by the current method for OTs have certain similarities with the previously reported methods. Our method highlights the ability to correct for strong matrix effects in capsicum substrates based on isotopic internal standards, as well as the environmental friendliness of using DES.

## 3. Experimental

### 3.1. Materials and Methods

LC-MS grade methanol (MeOH) and acetonitrile (ACN) were purchased from CNW ANPEL (Shanghai, China); formic acid, sodium hydroxide (NaOH), hydrochloric acid (HCI), magnesium sulfate (MgSO_4_) and sodium chloride (NaCI) are both obtained by CNW ANPEL (Shanghai, China); ultrapure water used throughout was obtained through a Milli-Q purification system (Millipore, Bedford, MA, USA). Raw materials for synthesizing deep eutectic solvents, including decanoic acid, undecanoic acid, menthol, octanoic acid, nonanoic acid, were purchased from Aladdin (Shanghai, China)

Standards of OTA (>98%) and OTB (>98%) were purchased from pribolab (Qingdao, China). Standards of ^13^C_20_ OTA (10.21 μg/mL) and ^13^C_20_OTB (10.21 μg/mL) in acetonitrile were also obtained from Probolab (Qingdao, China). A standard stock solution of 1 mg/mL concentration for each mycotoxin in methanol (MeOH) was prepared and stored at −20 °C in the dark. A working solution of OTA and OTB at a concentration of 0.1 μg/mL was prepared using an acetonitrile solution containing 1% formic acid. As needed, these stock solutions were diluted with acetonitrile to prepare working solutions. 

The capsicum samples were purchased from local markets in Guiyang city, and all the samples were processed as received.

### 3.2. Chromatographic and Mass Spectrometric Conditions of LC-MS/MS

The HPLC system (Nexera X2; Shimadzu Corporation, Kyoto, Japan) was controlled using the Labsolutions software, version 5.97. A C_18_ column from Waters Acquity UPLC HSST3 (50 mm × 2.1 mm i.d., 1.8 μm) was used for the separation. The column was heated up to 40 °C. The stepwise elution program was combined with the following mobile phase: A solution, 0.1% formic acid in the water, and B solution, acetonitrile. The mobile phase consisted of a 40% B solution for the first 1.5 min, followed by 95% B solution from 1.5 to 4.0 min, and 40% B solution from 4.0 to 5.0 min. The flow rate was 0.3 mL/min for the whole process. The outside of the injection needle in the HPLC system was rinsed with 50% methanol (CNW ANPEL) in water. The temperature of the LC autosampler was set to 15 °C.

The MS system control and data processing were carried out by Labsolution V 5.113 software from Shimadzu (Kyoto, Japan). The injection volume was set to 2 μL for each analysis. The main mass spectrometry parameters of ochratoxin in this study were shown in Table 5.

### 3.3. Preparation of Deep Eutectic Solvents

HBA and HBD were mixed in a certain molar ratio and then placed in a water bath at a controlled temperature of 80 °C with magnetic stirring for 20 min until a homogeneous and transparent liquid was obtained, and the successful synthesis of DES was indicated when the liquid was maintained by cooling at room temperature.

### 3.4. Dispersive Liquid–Liquid Microextraction Procedure (DLLME)

All collected capsicum samples were crushed in a pulverizer to powder and stored in polypropylene tubes at room temperature in the dark. In a 50 mL polypropylene centrifuge tube, 1 g (±0.005 g) of powdered sample was added, and 5 mL of purified water was added and vortexed for 2 min and left to equilibrate for 30 min. Then, 5 mL of acetonitrile containing 1% formic acid was added as the extractant and the tube was shaken briefly and then continued to be vortexed for 5 min. The extracting salt packet (4.0 g of anhydrous magnesium sulphate + 1.0 g of sodium chloride) was added to the above centrifuge tube and immediately shaken vigorously to prevent it from clumping. Then, the above centrifuge tube was vortexed for 2 min and centrifuged at 5000 rpm for 10 min, and finally, the clarified liquid was taken out from the above test tube and transferred into a glass reservoir bottle and used for the subsequent liquid–liquid microextraction work.

**Figure 5:**
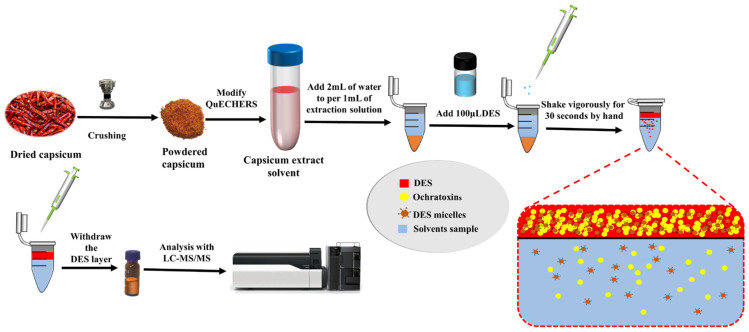
Schematic presentation of DLLME procedure using Thymol-based DES.

In a polypropylene test tube, 3 mL of pure water was added to every 1mL of capsicum extract. After that, 100 μL of DES solution was added and shaken vigorously by hand for 30 s, waiting for the solution to stratify, and the upper DES phase was aspirated using a pipette and loaded into the injection vial before entering the LC-MS system for analysis. The relevant operation is shown in Figure 5.

## 4. Conclusions

In this work, the DES based on thymol with decanoic acid was synthesized and evaluated as an extractant for the extraction of 2 OTs, i.e., OTA and OTB, from a modified QuECHERS method worthy pepper extract. The target analytes were separated and quantified by combining LC-MS/MS analytical methods. An exhaustive study was carried out in terms of the parameters affecting the extraction process to achieve good efficiency. The effects of the matrix were also attenuated by isotopic internal standardization, and the validation study successfully established a linear regression model fitting the experimental data (R^2^ ≥ 0.9905) and obtained relative recovery values in the range of 90–105% with an RSD ≤ 10% for all the target analytes in the chili extracts. Thirty chili pepper products commercially available in Guiyang City were also analyzed, respectively, and OTs contamination was found in five of them. Similarly, the greenness of the method was also assessed by both complexGAPI and AGREE methods, confirming that the developed method is in accordance with the principles of green analytical chemistry.

## Figures and Tables

**Table 1:** Preparation of the investigated alternatives of DESs.

Hydrogen Bond Donor (HBD)	Hydrogen Bond Acceptor (HBA)	Molar Ratio between HBD and HBA	DES Was Synthesized at Room Temperature	Images for the Prepared Mixtures
Decanoic acid	Thymol	1:1	Yes	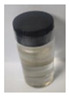
Decanoic acid	Thymol	1:2	No	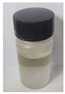
Nonanoic acid	Thymol	1:1	Yes	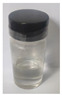
Menthol	Thymol	1:1	Yes	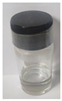
Undecanoic acid	Thymol	1:2	No	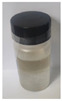
Nonanoic acid	Decanoic acid	1:1	Yes	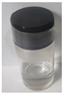
Octanoic acid	Thymol	1:1	Yes	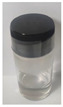
Undecanoic acid	Thymol	1:1	Yes	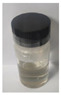
Menthol	Decanoic acid	1:1	Yes	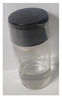

**Table 2:** Evaluation of matrix effects (ME%) under different methods (*n* = 3).

Parameter	Ochratoxins	Matrix Effects		Standard Curve		R^2^
		Standard Curve	Isotope Internal	External Standard Method	Isotope Internal Standard Method	
Matrix	OTA	−75.63	−2.22	y = 10837.32x + 5446.51	y = 0.83x + 0.05	>0.95
	OTB	−8.09	12.94	y = 29252.73x + 22367.98	y = 2.99x + 0.22	
DES	OTA	−71.29	3.16	y = 40871.50x − 4518.93	y = 0.96x + 0.0056	
	OTB	6.45	10.45	y = 108457.92x − 6646.02	y = 3.21x + 0.01	

**Table 3:** Analysis of real samples with OTs detected.

Sample Number	Sample Name	Detection of OTA μg/kg	Detection of OTB μg/kg
1	LJ-13	187.62	50.95
2	LJ-43	7.14	16.24
3	LJ-45	ND	8.24
4	LJ-49	ND	4.40

ND: Not detected.

**Table 4:** Comparison of various methods for detecting OTs.

Analytical Methods	Matrix	D.L. ^1^	Linear	Reference
HPLC-FLD	Coriander	0.05 µg/kg	10–51 μg/kg	[40]
ELISA	Capsicum	10 μg/kg	10–50 μg/kg	[41]
LC-MS-MS	Capsicum	0.15–0.45 μg/kg	0.5–250 μg/kg	This work

^1^ Limit of detection.

**Table 5:** Main mass spectral parameters of ochratoxins.

Compound	Precursor Ion	Product Ion	Collision Energy (eV)	Q1—Pre Deviation Voltage (eV)	Q3—Pre Deviation Voltage (eV)	Dwell Time (mSec)
OTA	403.8	238.95 *	−23	−12	−26	10
		357.95	−35	−12	−24	10
OTB	369.9	205.00 *	−21	−18	−22	10
		187.00	−34	−10	−12	10
$13_{C_{20}}$ OTA	423.9	250.00 *	−24	−12	−26	10
$13_{C_{20}}$ OTA	389.9	216.10 *	−21	−19	−23	10

* Product ion used for quantitative analysis.

## Data Availability

Data are contained within the article.
